# Phthalates in Glass Window Films of Chinese University Dormitories and Their Associations with Indoor Decorating Materials and Personal Care Products

**DOI:** 10.3390/ijerph192215297

**Published:** 2022-11-19

**Authors:** Liujia Fan, Lixin Wang, Kexin Wang, Fang Liu, Gang Wang

**Affiliations:** School of Environment and Energy Engineering, Beijing University of Civil Engineering and Architecture, Beijing 100044, China

**Keywords:** phthalates, window film, dormitory, indoor sources, consumer products

## Abstract

Phthalates are widely used as plasticizers in the production of various consumer products used daily. We analyzed phthalate concentrations in window film samples from 144 dormitories in 13 universities and combined them with the results of questionnaires to explore the associations of phthalate concentrations with indoor decorating materials and personal care products. The phthalate pollution levels discovered in this study were much higher than those in previous studies of baby rooms and university buildings. Moreover, it was found that phthalate concentrations in glass window films were associated with laminated wood or polyvinyl chloride (PVC) flooring, iron furniture, medium density fiberboard (MDF) furniture, and the usage frequency of bottled skincare products. Laminated wood or PVC flooring, wallpaper, and iron furniture are very likely sources of specific phthalates, and the large surface areas of MDF furniture can act as sinks of phthalates. Transport of phthalates from the packaging of bottled skincare products into cosmetics should be given more attention. Our results provide a deep understanding of the sources of phthalates in glass window films.

## 1. Introduction

Phthalic acid esters (PAEs) are currently the most widely used plasticizers and they accounted for 65% of the world plasticizer consumption in 2017 [1]. Numerous studies have reported detection of phthalates in a variety of products, such as building materials (wallpaper, synthetic leather, vinyl flooring and soft glass) [2,3,4], personal care products (face cream, lotions, perfumes, deodorants, hair care products, nail polishes and skin cleansers) [5,6,7], and other products (plastic bags, children’s toys, clothes, medicines and so on) [8,9,10,11].

Binding of phthalates to the product matrix involves noncovalent bonding [3], which easily releases phthalates from the product into the environment. Phthalates, which are endocrine-disrupting compounds, can enter the human body through dermal absorption, oral ingestion or inhalation and interact with a variety of cellular targets. Previous studies analyzed the possible binding efficiencies of phthalates and their metabolites on human estrogen receptors [12], the human peroxisome proliferator-activated receptor [13], and human ketosteroid receptors [14]. The results suggested that different patterns of phthalate interactions lead to different efficiencies of binding to receptors. Such phthalate binding eventually interferes with normal hormone secretion and causes health problems in humans. Exposure to phthalates could decrease thyroid hormone levels in children and pregnant women, causing hypothyroidism risk [15,16,17,18], as well as androgen levels [19,20] and follicle-stimulating hormone levels [21,22], causing fertility problems.

Phthalates in the environment can coexist in indoor multiphase media, such as air, particles, settled dust and surfaces [23]. Phthalate concentrations in indoor gas and particle phases reflect relative instantaneous concentrations, and indoor dust can accumulate phthalates [24]. Therefore, some studies have reported that indoor products were associated with the concentrations of phthalates in dust. Zhang et al. found a range of associations between perfume and diethyl phthalate (DEP) concentration: between plastic steel and butyl benzyl phthalate (BBzP) concentration; between laminated wood and the concentrations of di-iso-butyl phthalate (DiBP), BBzP and diisononyl phthalate (DiNP); and between leather polish and di(2-ethylhexyl) phthalate (DEHP) [25]. Bamai et al. reported that laminated wood floors were associated with DiBP concentrations and that PVC floors were associated with DEHP concentrations [26]. Kolarik et al. found associations between polishing agents and the concentrations of BBzP, DEHP and di-n-octyl phthalate (DnOP) [27]. Bornehag et al. reported that the amount of PVC used in wall coverings and floorings was associated with the concentrations of BBzP and DEHP [28]. Hung et al. reported associations between floor cleaning detergents and DEHP concentration [29].

Phthalate concentrations in indoor dust are easily affected by surfaces, such as surfaces of materials containing phthalates [26,30]. Therefore, the associations between long-term accumulation of phthalates and indoor products may not be well reflected. Studies have reported that organic films on impervious surfaces such as glass can be formed by gas-phase compounds and particle deposition from the air [31,32]. Organic films on indoor glass surfaces (referred to herein as glass window films) act as “natural” passive samplers that can accumulate phthalates via surface adsorption of airborne phthalates in indoor air by partitioning. Therefore, window films characterized by impermeability and uniformity can be used to reflect long-term phthalates pollution levels. Many materials and products are stored in university dormitories and measuring each one would require a very large amount of work. Thus, we can correlate indoor materials and products with phthalate concentrations in glass window films by a logistic regression model; this approach also contributes to understanding indoor pollution and identifying potential sources of indoor phthalates. However, no information has been reported on phthalates in glass window films and their associations with indoor decorating materials and personal care products.

The aim of this study is to determine phthalate concentrations in glass window films used in Chinese university dormitories and to explore the associations these concentrations have with indoor decorating materials and personal care products.

## 2. Materials and Methods

### 2.1. Standards

Fifteen phthalates were analyzed: dimethyl phthalate (DMP), DEP, DiBP, di-n-butyl phthalate (DnBP), di(2-methoxyethyl) phthalate (DMEP), di(4-methyl-2-pentyl) phthalate (DMPP), di(2-ethoxyethyl) phthalate (DEEP), dipentyl phthalate (DPP), di-n-hexyl phthalate (DnHP), BBzP, dicyclohexyl phthalate (DCHP), DEHP, DnOP and di-nonyl phthalate (DNP). Detailed information on the standard solutions is shown in Table S1 of the Supplementary Materials (SM).

### 2.2. Survey and Inspection

**Figure 1:**
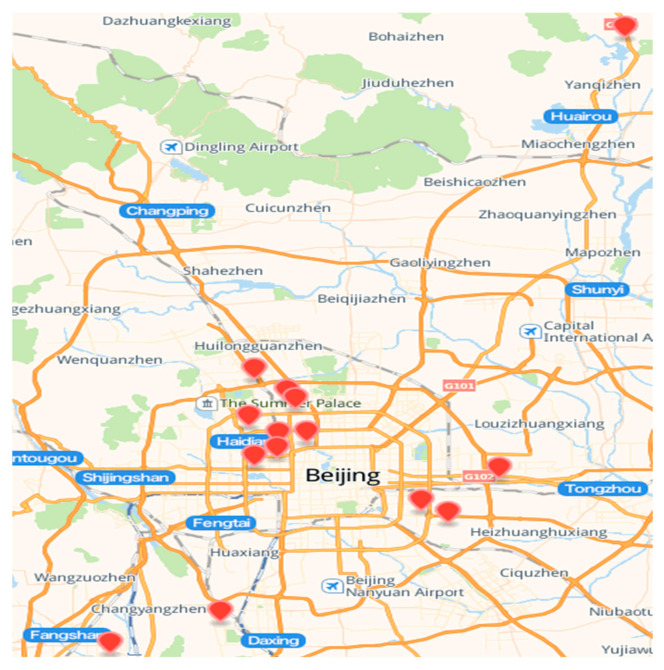
Sampling sites.

We sampled window films in 144 dormitories on 14 campuses of 13 universities in Beijing from May to December 2019. Figure 1 shows the sampling sites. Questionnaires were distributed to college students to gather information. This included basic information about their dormitory of residence, such as the university, building and dormitory number, room area, number of occupants, duration of occupancy and gender of occupants; dormitory interior decoration information, such as wall materials (emulsion paint and lime), floor materials (cement, tile, PVC boards and laminated wood), PVC wallpaper coverage and furniture materials (iron, MDF, leather and plastic) and quantity; the occupants’ living habits, such as the number of personal care products (liquid, milky, frothy and spray) per capita (fewer than 5 bottles, 5–10 bottles and more than 10 bottles) and frequency of use (never, 2–3 times per month, 2–3 times per week and at least one time per day), and frequency of use of cleaning products (washing liquid, sterilizing liquid and softener; 2–3 times per month, 2–3 times per week, and at least one time per day). A total of 230 questionnaires were issued, and the response rate was 84.3%. Then, the questionnaires were consolidated and sorted by dormitory as a unit. A total of 144 valid questionnaires were obtained.

### 2.3. Sample Collection

The sampling area was 20 cm × 20 cm, and the distance from the window gap was at least 10 cm and at least 0.5 m from the indoor floor. Airlaid paper soaked in dichloromethane (chromatography reagent, Mreda Technology Inc., Beijing, China) was wrapped on a Teflon flake and clamped to wipe the sampling area four times. The field blanks were sampled by taking the Airlaid paper from dichloromethane and waving it in the air for 10 s; it was then stored in a jar and sealed for storage in the laboratory at a low temperature and in the dark.

Samples were identified with the simultaneously recorded temperature, humidity and concentration of PM_10_. The indoor temperature and relative humidity were in the range of 16.4–28.9 °C and 14.6–69.6%, respectively, and the median was 21.6 °C and 35.1%, respectively. The indoor PM_10_ concentration was in the range of 29.2–557.6 μg/m^3^, and the median was 141.4 μg/m^3^. The outdoor temperature and relative humidity were in the range of 18–33 °C and 17–72%, respectively, and the median was 28 °C and 32%, respectively.

### 2.4. Sample Analyses

In our previous study, detailed information is provided on the pretreatment and analytical methods for glass window films [33]. Briefly, 10 mL of dichloromethane was poured into a jar to completely submerge the Airlaid paper, which was subjected to ultrasonic irradiation for 15 min (the extraction temperature was 25 °C). This step was performed three times, and the three extractions were collected into the same beaker. The extract was concentrated to 5 ml by rotary evaporation (70 °C, 50 rpm). The concentrated extract was reduced to 1 mL by bubbling with nitrogen. After filtration through a 0.45 μm microporous filter membrane, the volume was adjusted to 1 mL.

The concentrations of phthalates were determined by gas chromatography–mass spectrometry (7820 A-5977 E, Agilent Technologies Inc., Beijing, China). Detailed information on the analytical methods and detection limits is given in Section S2 of the SM.

### 2.5. QA/QC

Airlaid paper with dichloromethane was put into a Soxhlet extractor for extraction at 70 °C for 24 h and dried in a drying oven at 100 °C. All glassware was washed with dichloromethane and dried in a drying oven at 150 °C. Detailed information on the recovery rates, blanks, residuals and precision of the measurement method can be found in Section S3 of the SM.

### 2.6. Statistical Analyses

First, the concentrations of phthalates were not normally distributed according to the Shapiro–Wilk test. Therefore, the differences in phthalate concentrations in dormitories with or without decorating materials and personal care products were compared with the Mann–Whitney U analysis. Then, decorating materials and personal care products exhibiting significant association (*p* < 0.05) with phthalate concentrations were used in a univariate logistic regression analysis; multicollinearity among the factors was excluded. The variables in the univariate model that reached a significant level (*p* ≤ 0.2) were included in multivariate logistic regression analysis to identify the strongest associations among decorating materials and personal care products with phthalate concentrations (*p* < 0.05). Data analysis was conducted using SPSS (version 25.0).

## 3. Results

### 3.1. Survey Results

Table 1 shows the results of the questionnaires on decorating materials. Most dormitory floor materials contained lime and cement. Only 9% of dormitories had wallpaper. The furniture in the dormitory was mainly made of wood and iron; more than half of the dormitories had less than nine pieces of iron furniture and seven pieces of MDF furniture.

The results of the questionnaires on personal care products were compiled (Table 2). Only 21% of dormitories had 15 bottles or more of bottled skincare products, but the frequency of use was very high. Most dormitories had less than five bottles of spray skincare products, and more than half of the dormitory used them less than 2–3 times per week. Cleaning products were also used frequently in dormitories.

### 3.2. Phthalate Concentrations

Detection frequencies and concentrations of phthalates were determined (Table 3). Detection frequencies of the most common phthalates were 100%, and the median concentrations decreased in the following order: DCHP > DEHP > DnBP > DEEP > DNP > DnOP > DiBP. Detection frequencies of other phthalates were all below 100% and they were excluded from statistical analysis.

### 3.3. Associations between Decorating Materials and Concentrations of Phthalates

Table S2 of the SM presents the concentrations of phthalates in the glass window films with different decorating materials. Concentrations of DiBP, DCHP, DEHP and DnOP were higher in dormitories with materials containing plasticizers in the floor covering than in those without plasticizers in the floor covering (*p* < 0.05). Concentrations of DnBP, DCHP, DEHP and DnOP were higher in dormitories with PVC wallpaper than in other dormitories. Concentrations of DnBP, DnOP and DNP were lower in dormitories with many MDF furniture pieces. The DnBP concentration was higher in dormitories with many iron furniture pieces. Crude odds ratios of decorating materials are shown in Table S3. Furthermore, the adjusted odds ratios of these variables are shown in Table 4. The final analysis showed that the following four variables likely affected phthalate concentrations: floor covering type, PVC wallpaper covering, the number of MDF furniture items and the number of iron furniture items.

### 3.4. Associations between Personal Care Products and Concentrations of Phthalates

Table S4 of the SM shows the association between phthalate concentrations in glass window films and different personal care products. The median concentrations of DEEP were significantly higher in dormitories with many bottled skincare products. Concentrations of DEEP, DCHP, DEHP and DnOP were significantly higher in dormitories that regularly used bottled skincare products. DPP concentrations were significantly higher in dormitories with many spray skincare products. DnBP concentrations were significantly higher in dormitories that regularly used spray skincare products. Crude odds ratios of personal care products are shown in Table S5. Furthermore, the adjusted odds ratios of these variables are shown in Table 5. The final analysis showed that the frequency of consumption for bottled skincare products likely affected phthalate concentrations.

## 4. Discussion

Fourteen phthalate esters were detected (Table 3); detection rates ranged from 2% to 100%, and the median concentrations ranged from nd (not detected) to 3.53 × 10^2^ μg/m^2^. The most predominant phthalates in the dormitory window film samples were DnBP, DCHP and DEHP, and there are substantially fewer studies on phthalates in window films than on phthalates in house air and dust [34,35,36,37,38,39,40,41], which is similar to the case for previous study results on baby rooms and university buildings in China [42,43]. However, the median concentrations of DnBP and DEHP (1.75 × 10^2^ μg/m^2^ and 3.14 × 10^2^ μg/m^2^, respectively) in this study were significantly higher than those in baby rooms (85.3 μg/m^2^ and 48.2 μg/m^2^, respectively) and university buildings (winter: 1.5 μg/m^2^ and 7.6 μg/m^2^, respectively; summer: 1.3 μg/m^2^ and 3.6 μg/m^2^, respectively). Possible explanations for this observation are that dormitories are typical indoor environments on campuses, they are characterized by their small spaces and high population densities, and there are far more sources of phthalates in dormitories than in baby rooms and university buildings. On the other hand, the cleaning habits of university students were not good, and a relatively low frequency was indicated for window cleaning. In 69% of the dormitories we surveyed, the indoor window glass had not been cleaned since check-in. These two factors led to higher concentrations of phthalates in this study than in baby rooms and university buildings in China. Therefore, health problems of university students should be considered, and students should also change their cleaning habits and reduce the sources of phthalates to reduce phthalate pollution in dormitories.

We found a range of associations between floor materials with plasticizer and the concentrations of DiBP, DCHP, DEHP and DnOP. The floor materials in the dormitories containing plasticizers included laminated wood and PVC boards. Laminated wood floors are usually made from 4 to 5 thin pieces of laminated boards held together with adhesive and covered with paint [26], and the paints and adhesives utilized contain DiBP [44,45,46]. Analogously, PVC floor materials often add DCHP, DEHP and DnOP with high molecular weights to convert from hard plastic to elastic material with increased plasticity [47,48,49]. Therefore, indoor flooring materials are important sources of phthalates. Bamai et al. reported higher DiBP concentrations in the floor dust of homes with laminated wood floors and higher DEHP concentrations with PVC floors compared to other flooring materials [26]. Bornehag et al. found that high concentrations of BBzP and DEHP in dust were associated with PVC flooring [28]. These results further confirmed that phthalates can be dispersed into the environment from floor materials. Additionally, our results indicated that the low molecular weight that DiBP present in the gas phase can accumulate in window films, and DCHP, DEHP and DnOP with higher molecular weights are mainly present as particulates and can also accumulate in window films.

A strong association was found between the amount of iron furniture and the DnBP concentration. The main sources of phthalates in iron furniture are the paints or coatings used for large areas on the surfaces of the furniture, and the flexibility and adsorption force of paint and coating film can be strengthened to make the film firm and durable by adding plasticizers. DnBP is commonly used in lacquers, coatings and varnishes [45,50]. Zhao studied wall surface coating samples and found that DnBP was the most abundant phthalate, with a concentration of 17.2–26.6 μg/g [51]. Additionally, an association between PVC wallpaper and DnBP concentration was indicated in our study. Uhde et al. measured phthalate emissions from plastic wallpaper over 14 days and found that the highest DnBP concentration in indoor air due to plastic wallpaper was 5.1 μg/m^3^ [2]. Fujii et al. reported that DnBP was detected in PVC wallpaper [3]. Bamai et al. also reported that DnBP may be contained in materials such as paint, paper and wooden walls or ceilings [26]. We collected the paint films on the surfaces of iron furniture and wallpaper in the sampled dormitory and detected phthalates in those materials. The results showed that the DnBP concentrations in the paint film and wallpaper were 35.5 μg/g and 27.0 μg/g, respectively (Section S6 of the SM). In summary, indoor wallpaper and paint are the sources of DnBP in window films.

Large amounts of MDF furniture were significantly associated with low concentrations of DnBP, DnOP and DNP. MDF furniture is composed of wood fibers or other plant-fiber-based raw materials pressed into furniture. Some studies have suggested that furniture is an indoor source of phthalates and releases large amounts of phthalates into the environment [27,44,45]. In fact, large amounts of MDF furniture may also be sinks for phthalates. A prior study reported that porous materials acting as sinks accumulated indoor phthalates, and, when the ventilation was enhanced, the phthalates in the porous materials were released again, thereby reducing the effectiveness of ventilation [52]. Wood can act as a sink because of strong phthalate adsorption on the wood surface [53]. Furthermore, wood is a porous material and has a large internal adsorption area in addition to the surface adsorption area, and phthalates can also diffuse into wood [53,54]. Liang and Xu reported that the gas-phase concentrations of DEHP and DiNP decreased with increasing adsorption surface area in the wooden chamber [54]. More importantly, most of the dormitories we studied were occupied by four to six persons and had large amounts of furniture with surfaces oriented upward; most of this furniture was located some distance from the floor, which allowed particles from the air to accumulate on the upward-facing furniture [55]. The combined effects of these factors made MDF furniture the dominant sink for phthalates, and the concentrations of DnBP, DnOP and DNP in the glass window films decreased with increasing numbers of MDF furniture pieces.

We found a significant association between frequency of bottled skincare product use and concentrations of DEEP and DnOP. Conventionally, relatively low molecular weight phthalates are used in the manufacture of cosmetics to enhance the toughness and comfort of products and weaken the brittleness of the material [56,57,58]. Studies have been performed to test the ingredients in cosmetics. Among the five phthalates, DEP and DnBP were the phthalates most frequently detected in 102 cosmetic samples from retail stores in Seoul, Korea [5]. Five phthalates (DEP, DnBP, DiBP, DEHP and DMP) among the 18 phthalates were detected to some extent in 252 of the cosmetic samples collected from retail stores in Canada, while DEEP and DnOP were not detected [6]. DEP, DnBP, DiBP and DEHP were most frequently found among the nine phthalates present in 52 cosmetics collected from Tianjin, China, while DnOP was not detected [7]. Cosmetics safety issues are of great concern. In the 2007 edition of the Hygienic Standard for Cosmetics in China, DnBP, DEHP, DMEP, DPP and BBzP were listed as prohibited components in cosmetics [59]. The above study results differed from ours. The cosmetics reported were new cosmetics purchased from the market; however, the cosmetics sampled in our study had been used for a long time. Some studies reported that DnOP was detected in plastic packaging [8] and may also be present in bottle cap liners [50]. DEEP may be present in plastic packaging [49]. Therefore, we suppose that phthalates in packaging can be transported into cosmetics during use. Plastic packaging and bottle cap liners were very likely the sources of DnOP and DEEP in cosmetics and we should pay more attention to this. It is worth noting that DnOP and DEEP were not associated with the number of bottled skincare products used, and the reason for this requires further study.

In published studies on the associations between indoor products and phthalate concentrations in dust [25,26,27,28], some phthalates were not observed, such as DCHP, which may be harmful to humans. Wang et al. (2013) found the metabolite of DCHP (monocyclohexyl phthalate, MCHP) in the urine of 259 children in China, and found that MCHP was positively associated with body mass index and waist circumference, which may contribute to childhood obesity [60]. We analyzed fifteen phthalates: the predominant phthalate in the dormitory window film samples was DCHP (median concentration: 3.53 × 10^2^ μg/m^2^; detection frequency: 100%). Therefore, we consider these uncommon phthalates to be of concern. Moreover, phthalates can coexist in a variety of indoor environmental media; high-molecular-weight phthalates tend to exist in the particle phase, and low-molecular-weight phthalates are mainly in the gas phase [61], but the sources of phthalates in each medium may be different. Therefore, the associations between phthalates in environmental media and indoor materials and products are worth further study. Furthermore, correlating indoor materials and products with phthalate concentrations through binary logistic regression promotes the rapid understanding of indoor contamination and identification of potential sources of indoor phthalates.

## 5. Conclusions

The predominant phthalates found in glass window films of university dormitories in Beijing, China were DnBP, DCHP and DEHP. The phthalate pollution levels in this study were much higher than those in previous baby room and university building studies, due to the large number of sources and the low frequency of window cleaning in university dormitories. Extensive use of laminated wood or PVC flooring, PVC wallpaper and iron furniture, as well as high usage frequency for bottled skincare products in the dormitory increased the specific phthalate concentrations. Transport of phthalates from skincare packaging into cosmetics during use should be given more attention. It is of concern that a large amount of MDF furniture in the dormitory reduces the concentrations of certain phthalates due to the large upward-facing surface areas and adsorption effects. In our opinion, phthalates in dormitories should be studied extensively in the future, and university students should reduce the use of products containing large amounts of phthalates and improve their cleaning habits.

## Figures and Tables

**Table 1:** Decorating materials.

		Number of Samples (%)
Floor covering	Materials without plasticizer ^a^	104 (85%)
	Materials with plasticizer ^b^	18 (15%)
PVC wallpaper covering	No	111 (91%)
	Yes	11 (9%)
Amount of MDF furniture ^c^	<9 pieces	71 (58%)
	≥9 pieces	51 (42%)
Amount of iron furniture ^d^	<7 pieces	64 (52%)
	≥7 pieces	58 (48%)

^a^ Materials without plasticizers included tile and cement; ^b^ materials with plasticizers included PVC boards and laminated wood; ^c^ MDF furniture included wooden beds, wooden tables, wooden chairs and wooden cabinets; ^d^ iron furniture included iron beds, iron tables, iron chairs and iron cabinets.

**Table 2:** Personal care products.

		Number of Samples (%)
Number of bottled skincare products ^a^	Less (<15 bottles)	89 (79%)
	More (≥15 bottles)	24 (21%)
Frequency of bottled skincare products use	Low (<once per day)	36 (32%)
	High (≥once per day)	77 (68%)
Number of spray skincare products ^b^	Less (<5 bottles)	95 (84%)
	More (≥5 bottles)	18 (16%)
Frequency of spray skincare products use	Low (<2–3 times per week)	64 (57%)
	High (≥2–3 times per week)	49 (43%)
Frequency of cleaning products use ^c^	Low (<2–3 times per week)	10 (9%)
	High (≥2–3 times per week)	97 (91%)

^a^ Bottled skincare products were divided into liquid (such as toner, makeup remover), milky (such as lotion, essence) and frothy (such as face cream, vanishing cream); ^b^ spray skincare products included perfume, hair spray, freshener and so on; ^c^ cleaning products included washing liquid, sterilizing liquid, softener and so on.

**Table 3:** Detection frequency and concentrations of phthalates (μg/m^2^).

Phthalates	Detection Frequency, %	Mean	Min	Median	Max
DMP	52	22.39	nd	2.19	127.85
DEP	51	29.54	nd	56.96	98.64
DiBP	100	57.46	26.71	39.34	212.41
DnBP	100	206.13	38.56	174.93	811.86
DMEP	65	35.88	nd	40.87	209.49
DMPP	2	0.45	nd	nd	43.61
DEEP	100	195.74	31.98	75.71	1512.50
DPP	89	21.69	nd	26.98	88.50
DnHP	94	35.22	nd	14.75	360.62
BBzP	42	1.01	nd	nd	27.66
DCHP	100	433.15	51.44	353.30	2351.72
DEHP	100	425.35	34.54	314.16	2624.63
DnOP	100	68.81	31.13	56.27	455.89
DNP	100	118.80	41.38	64.97	1059.85

**Table 4:** Results of multivariate logistic regression analysis between decorating materials and phthalate concentrations (95% confidence interval).

		DiBP	DnBP	DEEP	DCHP
Floor covering	Materials without plasticizer	1.00	1.00	1.00	1.00
	Materials with plasticizer	30.41 (3.77~245.43) **	-	-	4.63 (1.35~15.90) *
PVC wallpaper covering	No	1.00	1.00	1.00	1.00
	Yes	-	16.18 (1.19~219.96) *	-	-
Amount of MDF furniture	<9 pieces	1.00	1.00	1.00	1.00
	≥9 pieces	-	0.33 (0.14~0.75) *	-	-
Amount of iron furniture	<7 pieces	1.00	1.00	1.00	1.00
	≥7 pieces	-	3.02 (1.32~6.86) *	-	-
		**DEHP**	**DnOP**	**DNP**	
Floor covering	Materials without plasticizer	1.00	1.00	1.00	
	Materials with plasticizer	4.52 (1.32~15.46) *	3.85 (1.05~14.08) *	-	
PVC wallpaper covering	No	1.00	1.00	1.00	
	Yes	-	-	-	
Amount of MDF furniture	<9 pieces	1.00	1.00	1.00	
	≥9 pieces	-	0.34 (0.15~0.78) *	0.30 (0.13~0.68) **	
Amount of iron furniture	<7 pieces	1.00	1.00	1.00	
	≥7 pieces	-	-	-	

“*” represents the *p*-value less than 0.05; “**” represents the *p*-value less than 0.01.

**Table 5:** Results of multivariate logistic regression analysis between personal care products and phthalate concentrations (95% confidence interval).

		DiBP	DnBP	DEEP	DCHP
umber of bottled skincare products	Less (<15 bottles)	1.00	1.00	1.00	1.00
	More (≥15 bottles)	-	-	-	-
Frequency of bottled skincare product use	Low (<once per day)	1.00	1.00	1.00	1.00
	High (≥once per day)	-	-	3.16 (1.19~8.40) *	-
Number of spray skincare products	Less (<5 bottles)	1.00	1.00	1.00	1.00
	More (≥5 bottles)	-	-	-	-
Frequency of spray skincare product use	Low (<2–3 times per week)	1.00	1.00	1.00	1.00
	High (≥2–3 times per week)	-	-	-	-
		**DEHP**	**DnOP**	**DNP**	
Number of bottled skincare products	Less (<15 bottles)	1.00	1.00	1.00	
	More (≥15 bottles)	-	-	-	
Frequency of bottled skincare product use	Low (<once per day)	1.00	1.00	1.00	
	High (≥once per day)	-	3.74 (1.43~9.81) **	-	
Number of spray skincare products	Less (<5 bottles)	1.00	1.00	1.00	
	More (≥5 bottles)	-	-	-	
Frequency of spray skincare product use	Low (<2–3 times per week)	1.00	1.00	1.00	
	High (≥2–3 times per week)	-	-	-	

“*” represents the *p*-value less than 0.05; “**” represents the *p*-value less than 0.01.

## Data Availability

The data that support the findings of this study are available in the Supplementary Materials of this article.

## Supplementary Materials

The following supporting information can be downloaded at: https://www.mdpi.com/article/10.3390/ijerph192215297/s1, Table S1 in S1: Phthalates in mixing standards, Table S2 in S4: Median concentrations of phthalates as related to different decorating materials (μg/m^2^), Table S3 in S4: Results of univariate logistic regression analysis between decorating materials and phthalate concentrations (95% confidence interval), Table S4 in S5: Median concentrations of phthalates as related to different personal care products (μg/m^2^), Table S5 in S5: Results of univariate logistic regression analysis between personal care products and phthalate concentrations (95% confidence interval); S2: Instrumental analysis; S3: QA/QC; S6: Sampling and measurement of indoor paint film and wallpaper.
