# Supplementary material for: Phthalates in Glass Window Films of Chinese University Dormitories and Their Associations with Indoor Decorating Materials and Personal Care Products
Source: Int J Environ Res Public Health. 2022 Nov 19;19(22):15297. doi: 10.3390/ijerph192215297 (PMC9696275; doi:10.3390/ijerph192215297)
Supplement: Supplementary file 1 [file ijerph-19-15297-s001.zip › ijerph-2016994-supplementary.pdf]

Supplementary Material

Phthalates in Glass Window Films of Chinese University Dormitories and Their Associations with Indoor Decorating Materials and Personal Care Products

Liujia Fan, Lixin Wang *, Kexin Wang, Fang Liu * and Gang Wang

School of Environment and Energy Engineering, Beijing University of Civil Engineering and Architecture, Beijing 100044, China
* Correspondence: wanglixin@bucea.edu.cn (L.W.); liufang@bucea.edu.cn (F.L.)

S1. Phthalate standards

Mixing standards with 15 kinds of phthalates (Cat. No. M-8061-R1, AccuStandard, USA, 1000 μg/mL) were used to calibrate the samples. The components of mixing standards are shown in Table S1.

Table S1. Phthalates in mixing standards.

Components	CAS No.	Formula	MW
Dimethyl phthalate (DMP)	131-11-3	$C_{10}H_{10}O_4$	194.18
Diethyl phthalate (DEP)	84-66-2	$C_{12}H_{14}O_4$	222.24
Di(isobutyl) phthalate (DiBP)	84-69-5	$C_{16}H_{22}O_4$	278.34
Di-n-butyl phthalate (DnBP)	84-74-2	$C_{16}H_{22}O_4$	278.34
Di(2-methoxyethyl) phthalate (DMEP)	117-82-8	$C_{14}H_{18}O_6$	282.29
Di(2-ethoxyethyl) phthalate (DEEP)	605-54-9	$C_{16}H_{22}O_6$	310.34
Dipentyl phthalate (DPP)	138-18-0	$C_{16}H_{24}O_4$	306.40
Butyl benzyl phthalate (BBzP)	85-68-7	$C_{19}H_{20}O_4$	312.36
Dicyclohexyl phthalate (DCHP)	84-61-7	$C_{20}H_{26}O_4$	330.42
Bis(4-methyl-2-pentyl) phthalate (BMPP)	146-50-9	$C_{20}H_{30}O_4$	334.45
Dihexyl phthalate (DnHP)	84-75-3	$C_{20}H_{30}O_4$	334.46
Di(2-n-butoxyethyl) phthalate (DBEP)	117-83-9	$C_{20}H_{30}O_6$	366.45
Di(2-ethylhexyl) phthalate (DEHP)	117-81-7	$C_{24}H_{38}O_4$	390.56
Di-n-octyl phthalate (DnOP)	117-84-0	$C_{24}H_{38}O_4$	390.56
Di-nonyl phthalate (DNP)	84-76-4	$C_{26}H_{42}O_4$	418.61

S2. Instrumental analysis

Phthalate concentrations in the samples were determined by gas chromatography-mass spectrometry (7820A-5977E, Agilent Technologies Co. Ltd., USA) with a 30 m×0.25 mm×0.25 μm

fused-silica capillary column (HP-5MS). The analysis was performed using the full scan mode. The carrier gas was Helium, and the flow rate was 2.0 mL/min. The temperature of injector was 280 °C, splitless injection. The oven temperature was programmed from 100 °C (held for 2.0 min) and raised to 300 °C at 10 °C/min (held for 5.0 min). The ionization source was EI, and the temperature was 250 °C; The ionization energy was 70eV.

Mixed standards were used to calibrate the samples. The standards were diluted to obtain seven concentrations: 0.1 μg/mL, 0.5 μg/mL, 1.0 μg/mL, 2.0 μg/mL, 5.0 μg/mL, 8.0 μg/mL, 10 μg/mL, 20 μg/mL, 50 μg/mL, and 75 μg/mL. The injection amount was 1.0μL. The linear correlation coefficients (R^2) of the standard curves were more than 0.9935. The instrument detection limits (IDLs) of phthalates were calculated from a signal-to-noise ratio of 3 and are in the range of 0.001-0.015 μg/mL. The method detection limits (MDLs) were determined from the IDLs and sampling area (20 cm×20 cm) and ranged from 0.025 $\mu g/m^2$ to 0.37 $\mu g/m^2$.

S3. QA/QC

Ten samples were randomly selected, and the phthalate residuals were measured using the same pretreatment and analysis methods as those used before. The results showed that the residuals were below the detection limit, and the extraction efficiency was close to 100%. Two solvent blanks, two laboratory blanks, two transportation blanks, and two field blanks were collected. All the blanks were analyzed with the same method as that used for the samples. The phthalate concentrations were below the detection limit. The recovery rates of the phthalates were determined by adding 5, 10, and 20 μg of the phthalate standard to 10 mL of dichloromethane, and the recovery rates ranged from 57.3–112.2% (a), 51.5–125.2% (b), and 66.3–103.4% (c), respectively. If the phthalate

concentration was less than or equal to 5 μg/mL, between 5 and 20 μg/mL, and greater than or equal to 20 μg/mL, we corrected it using recovery rates (a), (b), and (c), respectively. The precision of the measurement method was assessed by using replicate measurements (n=7) and 5 μg/mL, 10 μg/mL, and 20 μg/mL standard mixture solutions. The relative standard deviation (RSD) ranged from 2.0% to 10.0%. All the samples were quantified against a phthalate standard mix using a ten-point calibration curve. The calibration curves of individual phthalates were linear over a concentration range of 0.1 to 75.0 μg/mL ($R^2 \geq 0.9935$).

S4. Analysis of decorating materials

Table S2. Median concentrations of phthalates as related to different decorating materials (μg/m²).

		DiBP	DnBP	DEEP	DCHP	DEHP	DnOP	DNP
Floor covering	Materials without	3.52E+01	1.85E+02	5.91E+01	2.56E+02	2.41E+02	5.56E+01	6.71E+01
	Materials with plasticizer	8.35E+01	1.70E+02	8.30E+01	4.68E+02	4.08E+02	6.24E+01	6.46E+01
	p	**0.000**	0.74	0.675	**0.001**	**0.002**	**0.009**	0.098
PVC wallpaper covering	No	3.54E+01	1.75E+02	6.08E+01	2.85E+02	2.48E+02	5.57E+01	6.68E+01
	Yes	4.04E+01	3.28E+02	6.80E+01	4.87E+02	5.70E+02	6.73E+01	6.70E+01
	p	0.113	**0.000**	0.607	**0.004**	**0.003**	**0.001**	0.564
Amount of MDF furniture	< 9 pieces	3.64E+01	2.10E+02	5.83E+01	2.75E+02	2.48E+02	5.95E+01	6.83E+01
	≥ 9 pieces	4.02E+01	1.56E+02	8.37E+01	3.41E+02	2.98E+02	5.34E+01	5.84E+01
	p	0.163	**0.000**	0.183	0.203	0.362	**0.006**	**0.013**
Amount of iron furniture	< 7 pieces	3.74E+01	1.66E+02	5.83E+01	2.86E+02	2.49E+02	5.47E+01	6.60E+01
	≥ 7 pieces	3.65E+01	2.49E+02	7.75E+01	3.02E+02	2.79E+02	5.94E+01	6.72E+01
	p	0.068	**0.000**	0.67	0.467	0.682	0.05	0.236

Table S3. Results of univariate logistic regression analysis between decorating materials and phthalate concentrations (95% confidence interval).

		DiBP	DnBP	DEEP	DCHP
Floor covering	Materials without plasticizer	1.00	1.00	1.00	1.00
	Materials with plasticizer	**36.58(4.67~286.58)**	**0.49(0.17~1.35)**	**2.06(0.74~5.74)**	**6.34(1.94~20.66)**
PVC wallpaper covering	No	1.00	1.00	1.00	1.00
	Yes	2.77(0.76~10.02)	**9.82(1.22~79.33)**	0.98(0.28~3.40)	**4.22(1.06~16.78)**
Amount of MDF furniture	< 9 pieces	1.00	1.00	1.00	1.00
	≥ 9 pieces	**2.04(0.97~4.26)**	**0.22(0.10~0.48)**	**1.63(0.79~3.37)**	**1.66(0.80~3.46)**
Amount of iron furniture	< 7 pieces	1.00	1.00	1.00	1.00
	≥ 7 pieces	**0.60(0.29~1.24)**	**4.37(2.03~9.42)**	1.57(0.76~3.21)	0.64(0.31~1.33)

		DEHP	DnOP	DNP
Floor covering	Materials without plasticizer	1.00	1.00	1.00
	Materials with plasticizer	**6.08(1.87~19.8)**	**2.92(0.97~8.78)**	0.66(0.24~1.81)
PVC wallpaper covering	No	1.00	1.00	1.00
	Yes	**4.06(1.02~16.15)**	**11.35(1.40~91.66)**	1.06(0.30~3.67)
Amount of MDF furniture	< 9 pieces	1.00	1.00	1.00
	≥ 9 pieces	1.57(0.76~3.25)	**0.33(0.16~0.71)**	**0.32(0.15~0.68)**
Amount of iron furniture	< 7 pieces	1.00	1.00	1.00
	≥ 7 pieces	0.69(0.34~1.43)	**2.39(1.15~4.95)**	1.16(0.57~2.36)

Bold represents the *p*-value less than 0.2.

S5. Analysis of personal care products

Table S4. Median concentrations of phthalates as related to different personal care products (μg/m²).

		DiBP	DnBP	DEEP	DCHP	DEHP	DnOP	DNP
Number of bottled skincare products	Less（< 15 bottles)	3.54E+0	1.77E+02	5.83E+01	2.86E+02	2.51E+02	5.63E+01	6.68E+0
	More（≥ 15 bottles)	3.86E+0	2.23E+02	1.34E+02	3.58E+02	3.12E+02	5.90E+01	6.50E+0
	p	0.608	0.214	**0.045**	0.643	0.855	0.500	0.699
Frequency of bottled skincare products use	Low（< once per day)	3.54E+0	1.90E+02	4.26E+01	2.13E+02	1.89E+02	5.24E+01	6.92E+0
	High（≥ once per day)	3.68E+0	1.73E+02	8.49E+01	3.28E+02	2.91E+02	5.86E+01	6.47E+0
	p	0.336	0.081	**0.002**	**0.007**	**0.007**	**0.003**	0.662
Number of spray skincare products	Less（< 5 bottles)	3.54E+0	1.83E+02	5.98E+01	2.87E+02	2.53E+02	5.63E+01	6.61E+0
	More（≥ 5 bottles)	3.90E+0	1.77E+02	1.09E+02	3.19E+02	2.78E+02	5.76E+01	7.04E+0
	p	0.706	0.695	0.156	0.900	0.981	0.760	0.643
Frequency of spray skincare products use	Low（< 2-3 times per week)	3.58E+0	1.98E+02	5.42E+01	2.83E+02	2.55E+02	5.66E+01	6.82E+0
	High（≥ 2-3 times per week)	3.64E+0	1.65E+02	8.37E+01	3.04E+02	2.66E+02	5.58E+01	6.16E+0
	p	0.922	**0.038**	0.114	0.681	0.830	0.848	0.056
Frequency of cleaning products use	Low（< 2-3 times per week)	3.91E+0	1.87E+02	8.59E+01	2.66E+02	2.31E+02	5.67E+01	6.45E+0
	High（≥ 2-3 times per week)	3.53E+0	1.81E+02	6.80E+01	2.86E+02	2.51E+02	5.65E+01	6.61E+0
	p	0.280	0.638	0.856	0.600	0.764	0.847	0.304

Table S5. Results of univariate logistic regression analysis between personal care products and phthalate concentrations (95% confidence interval).

	Variables	DiBP	DnBP	DEEP	DCHP
Number of bottled skincare products	Less（< 15 bottles)	1.00	1.00	1.00	1.00
	More（≥ 15 bottles)	1.51(0.61~3.75)	1.56(0.62~3.93)	**2.94(1.14~7.60)**	1.62(0.65~4.01)
Frequency of bottled skincare products use	Low（< once per day)	1.00	1.00	1.00	1.00
	High（≥ once per day)	1.62(0.70~3.75)	**0.46(0.20~1.05)**	**3.12(1.33~7.35)**	**1.89(0.82~4.38)**
Number of spray skincare products	Less（< 5 bottles)	1.00	1.00	1.00	1.00
	More（≥ 5 bottles)	1.79(0.65~4.95)	0.83(0.30~2.27)	**2.07(0.74~5.80)**	1.20(0.43~3.32)
Frequency of spray skincare products use	Low（< 2-3 times per week)	1.00	1.00	1.00	1.00
	High（≥ 2-3 times per	1.06(0.49~2.27)	**0.39(0.18~0.83)**	**1.65(0.78~3.50)**	1.17(0.55~2.49)

	Variables	DEHP	DnOP	DNP
Number of bottled skincare products	Less (< 15 bottles)	1.00	1.00	1.00
	More (≥ 15 bottles)	1.54(0.62~3.82)	1.16(0.47~2.85)	0.89(0.36~2.20)
Frequency of bottled skincare products use	Low (< once per day)	1.00	1.00	1.00
	High (≥ once per day)	**2.00(0.86~4.62)**	**2.49(1.10~5.63)**	0.70(0.31~1.55)
Number of spray skincare products	Less (< 5 bottles)	1.00	1.00	1.00
	More (≥ 5 bottles)	1.15(0.42~3.17)	1.22(0.44~3.37)	1.17(0.43~3.23)
Frequency of spray skincare products use	Low (< 2-3 times per week)	1.00	1.00	1.00
	High (≥ 2-3 times per week)	1.10(0.52~2.33)	0.85(0.40~1.78)	**0.51(0.24~1.09)**

Bold represents the *p*-value less than 0.2.

S6. Sampling and measurement of indoor paint film and wallpaper

We selected the dormitory where window film had been collected as our survey object, which is located in a dormitory of Daxing university campus in Beijing. A clean knife was used to collect the paint on the surface of iron furniture and wallpaper. The collected paint and wallpaper (weigh 0.1g) were sealed in aluminum foil bags. The samples were stored in a refrigerator at -20 °C after sampling and were pretreated within one week.

The pretreatment and analysis methods of paint film and wallpaper are the same as window films, which have been described.
